# Temporal dynamics of ecological networks: deciphering changes in cladoceran assemblages over the past ~ 150 years in response to land-use development

**DOI:** 10.1093/plankt/fbaf047

**Published:** 2025-09-20

**Authors:** Jennifer Pham, Zofia E Taranu, Madeleine E Aucoin, Zoë Rabinovitch, Cindy Paquette, Beatrix E Beisner, Irene Gregory-Eaves

**Affiliations:** Department of Biology, McGill University, 1205 Dr. Penfield Ave, Montréal, Québec, H3A 1B1, Canada; Groupe de recherche Interuniversitaire en Limnologie (GRIL), 1375 Thérèse-Lavoie-Roux Ave, Montréal, Québec, H2V 0B3, Canada; Groupe de recherche Interuniversitaire en Limnologie (GRIL), 1375 Thérèse-Lavoie-Roux Ave, Montréal, Québec, H2V 0B3, Canada; Aquatic Contaminants Research Division, Environment and Climate Change Canada, 105 McGill Street, Montréal, Québec, H2Y 2E7, Canada; Department of Biology, McGill University, 1205 Dr. Penfield Ave, Montréal, Québec, H3A 1B1, Canada; Bieler School of Environment, McGill University, 3534 University Street, Montréal, Québec, H3A 2A7, Canada; Department of Biology, McGill University, 1205 Dr. Penfield Ave, Montréal, Québec, H3A 1B1, Canada; Groupe de recherche Interuniversitaire en Limnologie (GRIL), 1375 Thérèse-Lavoie-Roux Ave, Montréal, Québec, H2V 0B3, Canada; Groupe de recherche Interuniversitaire en Limnologie (GRIL), 1375 Thérèse-Lavoie-Roux Ave, Montréal, Québec, H2V 0B3, Canada; Aquatic Contaminants Research Division, Environment and Climate Change Canada, 105 McGill Street, Montréal, Québec, H2Y 2E7, Canada; Département des Sciences de l’Environnement, Université du Québec à Trois-Rivières, 351 Bd des Forges, Trois-Rivières, Québec, G8Z 4M3, Canada; Département des Sciences Biologiques, Université du Québec à Montréal, 141 Président-Kennedy Ave, Montréal, Québec, H2X 1Y4, Canada; Groupe de recherche Interuniversitaire en Limnologie (GRIL), 1375 Thérèse-Lavoie-Roux Ave, Montréal, Québec, H2V 0B3, Canada; Département des Sciences Biologiques, Université du Québec à Montréal, 141 Président-Kennedy Ave, Montréal, Québec, H2X 1Y4, Canada; Department of Biology, McGill University, 1205 Dr. Penfield Ave, Montréal, Québec, H3A 1B1, Canada; Groupe de recherche Interuniversitaire en Limnologie (GRIL), 1375 Thérèse-Lavoie-Roux Ave, Montréal, Québec, H2V 0B3, Canada

**Keywords:** co-occurrence network, zooplankton, land-use, paleolimnology, community ecology

## Abstract

Ecological networks offer a comprehensive view of communities by capturing potential species interactions. While valuable for studying ecological change in the Anthropocene, many studies lack data across expansive temporal and spatial gradients. We addressed this gap by applying network approaches to paleolimnological records capturing strong land-use changes. We analyzed cladoceran assemblages, key aquatic organisms with identifiable subfossils, using two paleolimnological methods: (i) top-bottom comparisons of sediment records from 101 Canadian lakes with varying land-use intensity, and (ii) high-resolution core records from two impacted lakes in eastern Canada. We used correlation matrices of taxon relative abundances to calculate network metrics across land-use types and time periods. We found that lake communities currently experiencing high human impact changed through time, showing a decrease in connectance (proportion of realized to potential links) and an increase in modularity (measure of network subcommunities); these patterns were also observed in our full core analyses as well as in our randomized simulation exercise. Overall, this first pan-Canadian study of zooplankton paleo-networks provides new insights into how lake food webs have changed over a period of accelerated anthropogenic change.

## INTRODUCTION

Over the last decade, land-use change and other human activities have greatly altered many ecosystems, which led to shifts or declines in biodiversity (Rittenhouse *et al.,* 2010; Dornelas *et al.,* 2014; Murphy and Romanuk, 2014; Newbold *et al.,* 2015). In freshwater systems, watershed land-use can increase inputs of nutrients from agriculture (Taranu and Gregory-Eaves, 2008) and forest cover loss (Kreutzweiser *et al.,* 2008), salts from urbanization (Dugan *et al.,* 2017), and metal contamination from mining (Winegardner *et al.,* 2017); all of which can degrade water quality and impact aquatic communities. These community effects are typically measured by quantifying diversity changes over time or across sites along gradients of land-use intensity (e.g. Su *et al.,* 2021; Paquette *et al.,* 2022). A more nuanced understanding of community dynamics may be derived by considering observed or potential species interactions through ecological network analyses (McCann, 2007; Jordano, 2016). Ecological networks represent biotic interactions or associations between species in a community, where the nodes represent the species and the links represent their potential interactions. Networks also provide a key bridge between community structure and ecosystem functioning (McCann, 2007; Harvey *et al.,* 2017), and may be useful in identifying reorganization or altered species interactions following disturbances.

**Fig. 1:**
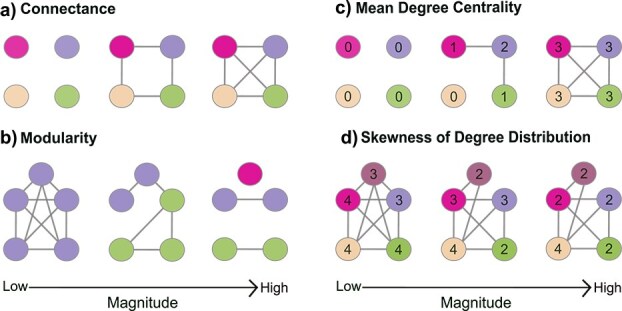
Diagram showing examples of networks with low to high values of selected network metrics. (**a)** Connectance is the proportion of realized and potential links. The colour of each node represent a taxon. As the network is more connected with links, connectance increases. (**b)** Modularity is the measure of subcommunities in a network. The colour represents the subcommunity that the nodes belong to. The higher the value, the more subdivided the network. (**c)** Mean degree centrality is the average number of links each node has. The number inside each node represents the number of links attached to the node. As each node is connected to more links, the mean degree centrality of the network increases. (**d)** Skewness of degree distribution measures how skewed the degree distribution is, alluding to how off-centre the ratio of nodes with high and low degree centrality is. From left-to-right, the networks with negative skewness tend to have nodes with greater degree centrality, while a network with positive skewness tend to have more nodes with lower degree centrality.

Environmental shifts resulting from human activities have been shown to alter ecological networks through various parameters. For instance, Wang *et al.* (2019) who conducted analyses on diatom assemblages from lakes with extensive upstream human disturbances, observed a negative change in skewness of degree distribution, which measures the frequency distribution in number of links (or associations) for each species (Fig. 1d). Negative skewness in degree distribution can provide evidence for generalist species (species with many links) dominating over time (Wang *et al.,* 2019). In networks of plankton and fish communities, climate change and nutrient enrichment have also been associated with lowered connectance (Fig. 1a), which is the proportion of realized to potential links (Merz *et al.,* 2023; Bonnaffé *et al.,* 2024). Network metrics can also be more sensitive for detecting human disturbance effects than diversity metrics, suggesting that the approaches are complementary (Li *et al.,* 2019). For instance, using a paleolimnological approach, Mayfield *et al.* (2022) demonstrated that network analyses based on subfossil chironomid assemblages could be more sensitive than beta-diversity metrics to measure taxon gain over time. Indeed, there has been a recent effort to study communities using both general diversity metrics and network ecology (e.g. Blackman *et al.,* 2022; Yu *et al.,* 2025). Finally, network analyses may be used to evaluate remediation efforts and best management practices, as a survey of stream food web networks showed that agriculturally-intensive watersheds can maintain their structure when buffered by riparian zones (Champagne *et al.,* 2022).

In light of their sensitivity to ecological disturbances, network metrics are increasingly used not only to track community change, but also to infer community stability (McCann, 2000; Landi *et al.,* 2018). For instance, connectance has become a key metric to describe network stability, contributing to the ongoing diversity-stability debate (e.g. Dunne *et al.,* 2002; Dunne *et al.,* 2004; Landi *et al.,* 2018). Dunne *et al.* (2002) found that independent of ecosystem type, simulated networks with higher connectance were more robust to extinction cascades. Modularity, a network metric measuring the compartmentalization of networks into groupings of highly connected species (Fig. 1b), was also postulated to contribute to stability because higher compartmentalization can inhibit effects of disturbances cascading through the network (May, 1972; Scheffer *et al.,* 2012). Species degree centrality (i.e. the number of links with others in the network), may be representative of the compositional persistence, as those with higher number of links have a higher chance of remaining connected with other species as disturbances removes links (Landi *et al.,* 2018). Often, mean degree centrality is calculated as a synthetic metric of species degree for the whole network (Fig. 1c). Overall, network ecology is a promising tool to assess the effects of human disturbances such as land-use, as well as infer community stability and complement traditional diversity indices.

There has been tremendous growth in network ecology (Lau *et al.,* 2017), yet key gaps remain. In aquatic ecosystems, robust observations of direct interactions are rarely possible; instead, researchers commonly use gut content analysis or stable isotopes to infer trophic interactions (e.g. Rawcliffe *et al.,* 2010; Champagne *et al.,* 2022, respectively). An alternative is to infer network links from correlations of co-occurrences or abundances across sites or time. This method does not require direct observations of interactions (e.g. competition or predation), making it simpler to apply to community presence-absence or abundance data. Furthermore, co-occurrence networks have been shown to detect known biotic interactions with moderate sensitivity and specificity (Freilich *et al.,* 2018). Indeed, several recent network studies based on co-occurrences have greatly advanced our understanding of aquatic community dynamics in the face of global changes (Wang *et al.,* 2019; Merz *et al.,* 2023; Yu *et al.,* 2025). Overall, co-occurrence analyses provide an important starting point for identifying changes in species associations due to interactions.

**Fig. 2:**
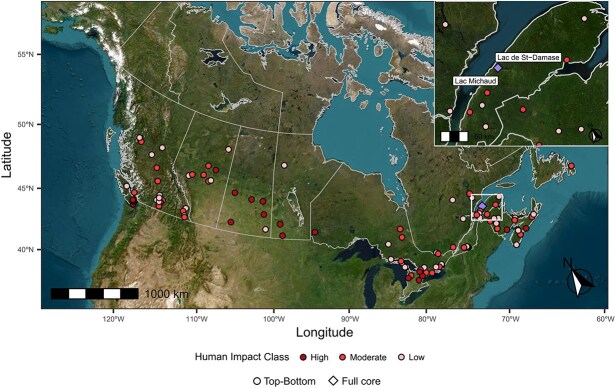
Map of study lakes in Canada. The circled points refer to lake sites where pre-Industrial and modern (i.e. bottom and top) intervals of the sediment cores were used to identify and compare the cladoceran assemblage (n = 101) from Paquette *et al.* (2022). The diamond points (overlapping due to their close proximity) are the full core study lakes, of which the cladoceran assemblage was identified throughout the length of the core (n = 2). Also depicted are the human impact class of the lakes in the top-bottom comparisons: high (n = 19), moderate (n = 39), low (n = 43). Lac de St-Damase has a human impact index (HII) of 0.21, while the HII for Lac Michaud is 0.42.

Another key knowledge gap is the lack of network studies with extensive spatial and temporal scales (i.e. across many sites and over more than a decade), which are relevant to understanding accelerating global environmental changes. By studying systems at continuous or multiple discrete time periods, we can detect network changes through time. In addition, as many sites may not have intense land-use nearby or may react to effects of land-use differently than others, we must also study multiple sites across broad regions to capture this potential heterogeneity. Herein, our goals were to describe patterns in network changes across many lakes within different land-use groups, and to explore how network metrics complement traditional diversity metrics, such as Shannon diversity, by extracting additional insights from our datasets. Therefore, we leveraged extensive paleolimnological datasets across spatial and temporal land-use gradients to address these objectives. Paleolimnological researchers often compare core top (i.e. modern) and core bottom (i.e. pre-Industrial) sediment intervals from any one lake core to enable the development of a larger dataset that spans many lakes across a landscape gradient of interest (Smol, 1992). Sediment cores can also be analyzed to quantify changes at a higher temporal resolution (i.e. time series from modern to pre-Industrial periods). For our analyses, we analyzed a 101-lake top-bottom dataset (Fig. 2), where sites were sampled as part of the NSERC Canadian Lake Pulse Network (LakePulse; Huot *et al.,* 2019). We complemented these data with analyses of two long-term sediment core records from eastern Canada, where each record spanned at least the last ~ 150 years (Fig. 2). The top-bottom approach allowed us to compare ecological networks between two disparate time-points from a large set of Canadian lakes and across a gradient of modern watershed land-use intensity, while the full core approach allowed us to detect shifts in ecological networks within a site through time. We used cladoceran zooplankton remains preserved in the sediment record as the observations of zooplankton species present in the lake at different time periods. Cladocerans are ideal organisms for studying ecological interactions due to their central position in the food web, both as grazers of primary producers and as prey for higher consumers (Jeppesen *et al.*, 2001). Their use also allowed us to build on the work of Paquette *et al.* (2022), who examined diversity changes in the modern and pre-Industrial subfossil cladocerans assemblages from the same 101 lakes across Canada. We hypothesized that sediment samples representing periods or sites with more intense human activity would show shifts in cladocerans network parameters relative to less disturbed lake watersheds. Specifically, we expected that disturbances related to human activity would lead to loss of links and species, possibly leading to decreases in connectance and skewness of degree distribution, but increased modularity and mean degree centrality.

## MATERIALS AND METHODS

### Site selection and field sampling

We used a top-bottom cladoceran assemblage dataset analyzed by Paquette *et al.* (2022) as well as two full cores of lakes in Quebec (Aucoin *et al.,* 2025)—all data were collected as part of LakePulse (Huot *et al.,* 2019). LakePulse uniformly sampled 663 pan-Canadian lakes across 12 ecozones during the summers of 2017–2019 (Huot *et al.,* 2019). Lake selection followed a stratified random sampling design considering lake ecozone, lake size, and a human impact index (henceforth referred to as HII). Lake size was determined by surface area, where small lakes were between 0.1–0.5 km^2^, medium-sized lake had 0.5–5 km^2^, and large lakes were 5–100 km^2^ (Huot *et al.,* 2019). The HII of each lake’s watershed was represented by a score between 0 and 1 quantifying anthropogenic land-use based on land-use type fractions (mining, agriculture, pasture, urban, natural landscape). Briefly, each 30 m pixel in the lake’s watershed was assigned a score between 0 and 1 based on the dominant fraction of land-use type, where agriculture, urban, and mines/oil were assigned a score of 1; pasture and recent forest clearcuts were 0.5; and natural landscape a 0. Each pixel value was then averaged together for the entire watershed, resulting in the HII. HII of less than or equal to 0.1 were “low” impact sites, between 0.1–0.5 were “moderate” impact sites, and HII greater than or equal to 0.5 were “high” impact sites (Paquette *et al.,* 2022; Fig. S1). Sediment cores were retrieved using a gravity corer from the deepest point or from a deep basin in each lake, which was determined by bathymetric maps or depth finders (Paquette *et al.,* 2022).

A subset of 101 LakePulse lakes, spanning nine ecozones, were selected based on the same overall stratified sampling design for determining subfossil cladoceran assemblages for the top and bottom intervals of the sediment cores (Paquette *et al.,* 2022; Fig. S2). This dataset consisted of two snapshot samples: modern (top of core) and pre-Industrial (bottom of core) conditions. All sediment samples were extruded on-site at 1-cm intervals using a vertical extruder. Confirmation that the deep sample reached the pre-Industrial period (pre-1850 CE) was done through ^210^Pb dating for a subset of 87 cores and literature analyses of regional lake sedimentation rates (Baud *et al.,* 2021; Paquette *et al.,* 2022).

We complemented the network analyses of the top-bottom dataset with parallel analyses of complete time series from two lakes. We analyzed full sediment cores of Lac de Saint-Damase (Lac de St-Damase), Quebec (48.65, −67.81) and Lac Michaud, Quebec (48.60, −67.82)—both of which have been previously studied in Aucoin *et al.* (2025) using a multi-proxy approach to infer effects of watershed land-use and climate change. The study lakes are both located within ~ 6 km from each other in the Atlantic Highland ecoregion. Lac de St-Damase has a lake area of 0.66 km^2^, a maximum depth of 3.4 m and a HII = 0.21. Lac Michaud has a lake area of 0.41 km^2^, a maximum depth of 5.7 m and a HII = 0.42. Full cores were stabilized with sodium polyacrylate powder at the lake shore, stored in a cooler, and then shipped overnight to Quebec City, where they were split longitudinally (see NSERC Canadian Lake Pulse Network, 2021). Cores were then subsampled at 1-cm intervals (see details in Baud *et al.,* 2023 and Aucoin *et al.,* 2025). At least 15 intervals for each core were prepared for ^210^Pb dating using gamma well detectors. The constant rate of supply (CRS) model was the best fitting chronological model for both lakes as determined by Baud *et al.* (2022).

### Lab methods

All sediment samples were freeze-dried prior to processing. To characterize the cladoceran assemblages over time, all sediment samples were digested following a slightly-modified methodology from Korhola and Rautio (2001). Briefly, between 0.1 and 0.2 g of freeze-dried sediments were digested in 10% potassium hydroxide solution over a hot-plate for 30 minutes. The mixture was then rinsed and sieved through a 36 μm sieve (not 50 μm as in the original method) with deionized water. The cladoceran solution was then transferred into a vial with a few drops of ethanol for preservation and glycerin-safranin dye solution for colour. About 1–5 aliquots of 80 μm of digested sediment was mounted on slides, with a glycerin-safranin dye mixture as the mounting medium. Subfossil cladocerans were identified using a DM 2500 Leica microscope under 200x-400x magnification. A single taxonomist (Cindy Paquette) identified all top and bottom samples; additional taxonomists (Madeleine E. Aucoin, Zoë Rabinovitch) calibrated their counting and identification abilities with this original dataset before processing the full cores. Several taxonomic keys were used (Szeroczyfiska and Sarmaja-Korjonen, 2007; Korosi and Smol, 2012a, 2012b). *Bosmina longirostris* and *Bosmina longispina* were distinguished based on the location and shape of the lateral headpore—as described in Korosi and Smol (2012b). Counts for *Bosmina coregoni* were combined with *B. longispina*. *Daphnia spp.* included indistinguishable subfossils of the *Daphnia longispina* and *Daphnia pulex* complex. Full coverslips were scanned for identifiable subfossil cladocerans. A minimum of 100 individuals were identified per depth interval (Kurek *et al.,* 2010). The counting effort across the intervals for both full cores ranged from 114 to 599 individuals.

### Statistical analysis

#### Network construction

All analyses in this study were conducted in R (v4.3.2; R Core Team, 2023). Network construction followed the method outlined in Barberán et al. (2012). Lakes from top-bottom analyses were subset according to their HII; high (n = 19), moderate (n = 39), and low (n = 43). Given that over the last ~ 150 years, there has been extensive documentation of species invasions, climate change, and other widespread global change which may shape species’ interaction or associations over time—we opted to develop separate networks for top and bottom communities over the land-use classes, leading to the construction of six networks. Subfossil count data was first transformed into relative abundances using the *cladCount* function from the *jezioro* package (Jeziorski, 2020), and then Hellinger-transformed (square root of relative abundances) as recommended prior to multivariate analyses (Legendre and Gallagher, 2001) using the *decostand* function from the *vegan* package (Oksanen *et al.,* 2022). The relative abundances of each site-by-species (for the top-bottom comparison) were used to compute the Spearman’s correlation coefficient of all possible species pairs (*rcorr* function, *Hmisc* package; Harrell, 2023). A strong correlation coefficient between two species (two columns of a matrix) reflects a similarity in their Hellinger values across sites (i.e. the matrix rows). In each network, nodes represented the taxa, and the edges represented the significant (*P* < 0.01) Spearman correlation coefficient between each taxa pair.

For the full cores, we first truncated the record to ~ 1750 CE to ensure coverage of a similar time period. Subfossil cladoceran relative abundances were Hellinger-transformed prior to analyses. We then identified time periods over which distinct cladoceran assemblages were observed by applying a constrained hierarchical clustering approach to each time series. To do so, we first applied the function *vegdist* from the *vegan* package (Oksanen *et al.,* 2022) to the full core cladoceran assemblages to calculate a percentage difference (aka Bray–Curtis) dissimilarity matrix. We then used the *chclust* function to realize a CONstrained Incremental Sum of Squares (CONISS; Grimm, 1987) cluster analysis, using the *rioja* package (Juggins, 2023). The validity of the clusters was checked with a broken stick model using the function *riojaPlot* and its argument *addRPClustZone* within the *riojaPlot* package (Juggins, 2023). Within the defined zones, we ensured a minimum of five intervals as this would allow for the creation of a Spearman correlation-based network. In contrast to the top-bottom networks (which relied on site-by-species matrices to compute significant Spearman correlation among taxa pairs), the paleo-networks were restricted to the sediment intervals found within each distinct cluster, hence depth-by-species matrices were used to calculate the Spearman correlations. Additionally, the significant Spearman correlation coefficient between taxa relative abundances with a *P* < 0.05 was chosen for robust links, as network structures within the same lake were being compared and the taxa pool was smaller. Lastly, the Shannon diversity index of all datasets was calculated to parallel the work of Paquette *et al.* (2022). We calculated Shannon diversity with the *diversity* function on the rarefied counts using the *rrarify* function (both functions are part of the *vegan* package; Oksanen *et al.,* 2022). For the top-bottom comparisons, we rarefied all counts to 93 individuals prior to calculating Shannon diversity. The cladoceran counts for the three most recent intervals of Lac de St-Damase were combined prior to rarefaction, so these binned intervals would encapsulate ~ 40 years, which represents a time span equivalent to that represented in the deeper 1 cm intervals. This was not done for Lac Michaud, as each interval represented ~ 7.5–9 years. Counts from the Lac de St-Damase full core were rarefied to 114 individuals whereas for Lac Michaud counts were rarefied to 136 individuals. For the full cores, the Shannon diversity values were then grouped by zone (as determined by the cluster analysis) and a Mann–Whitney–Wilcoxon test was performed using the *wilcox.test* function to test for significant differences in rarefied Shannon diversity among zones (full core analyses) or among temporal periods (top-bottom comparison).

#### Network parameters

Network parameters were chosen given their pertinence for inferring community stability in the literature (Landi *et al.,* 2018; Wang *et al.,* 2019; Li *et al.,* 2022). In particular, using the *igraph* package (Csárdi *et al.,* 2024), we calculated connectance (*edge.density* function), modularity (*edge.between.community* function followed by *modularity* function), species degree centrality (*degree* function), and skewness (Fig. 1). To visualize the networks, we constructed chord diagrams using the *circlize* package, where the links represent significant Spearman correlation coefficients and link width, the relative magnitude of the correlation coefficients (Gu *et al.,* 2014).

Since the networks constructed were not directional (no *a priori* defined taxa interactions), connectance was calculated as:


(1) \begin{equation*} \frac{L}{S\left(\frac{S-1}{2}\right)} \end{equation*}

where *L* is the total number of links, and *S* is the total number of species present in the network.

To calculate modularity, a community detection algorithm was employed which optimizes the division of the network into subgroups. We used the *edge.betweenness.community* function from the *igraph* package, which iteratively removes the network edges with highest network betweenness (i.e. edges that are identified as being between, rather than within, communities) and recalculating until subgroups were created (Newman and Girvan, 2004). The list of subgroups was then used to calculate modularity following:


(2) \begin{equation*} \frac{1}{2L}{\sum}_{i,j}\kern0em \left({A}_{ij}-\gamma \frac{k_i\kern0em {k}_j}{2L}\kern0em \right)\delta \left({c}_i,{c}_j\kern0em \right) \end{equation*}

where *L* is the number of edges, *A* is the adjacency matrix (i.e. species-by-species matrix) with row *i*, and column *j*, *k* is the degree of *i* and *j*, and *c* is the subdivision to which *i* and *j* belong according to the community detection algorithm.

Mean degree centrality was calculated as:


(3) \begin{equation*} \overline{X}=\frac{1}{S}{\sum}_{i=1}^S{l}_i \end{equation*}

where *l* is the number of links connected to species *i*, and *S* is the total number of species present in the network.

Skewness was calculated using the *skewness* function through the *moments* package (Komsta and Novomestky, 2022) and applied to the degree distribution. The skewness of the degree distribution was calculated as:


(4) \begin{equation*} \frac{\sum_{i=1}^S{\left({X}_i-\overline{X}\right)}^3\ }{\left(S-1\right){\sigma}^3} \end{equation*}

where *S* is the total number of species present in the network, *X* is the number of links connected to species *i*, $\overline{X}$is the mean degree centrality, and σ is the standard deviation of the degree distribution.

#### Random simulations of observed networks

Because we can only calculate one network for each group of samples, we created random configurations based on the observed network parameters to evaluate whether the network parameters for lakes in each HII class shifted significantly between modern (top) and pre-Industrial (bottom) periods. We also tested whether the network parameters for each full core lakes have shifted significantly over the stratigraphic zones. Random networks were constructed according to the Erdős-Rényi model, using the *erdos.renyi* function from the *igraph* package (Csárdi *et al.,* 2024). We constrained the random networks by the total number of species, *S*, present in one time slice and human impact class (or within the zone for the full core analyses), and the probability *p* of the occurrence of an edge between each node (i.e. the connectance). These values were derived from the constructed networks (i.e. the observed values). The random networks were constructed by connecting labeled nodes randomly and edges assigned with a probability *p*. Here, the assumptions of the model include that the edges are independent and are equally likely to occur. Network metrics listed previously (summarized in Fig. 1) were calculated for each random network created. To reflect the mean number of lakes in each HII class, 30 random networks were created for lakes grouped in their respective human impact class between modern and pre-Industrial periods. The same number of random networks were also constructed for each full core to be conservative as each core had at least 10 intervals. To determine whether the network parameters differed through time, we ran a one-way ANOVA (*aov* function) for each network parameter comparing time periods (tops with bottoms or among zones for the full cores) followed by a Tukey post-hoc test (*TukeyHSD* function). These statistical tests were applied separately to the random network generated for lakes categorized into low, moderate, and high HII for the top-bottom analyses, while the tests were applied separately for each full core. Normality and homoscedasticity assumptions were visually verified using the ANOVA model diagnostics in R. In addition, a p-value adjustment for multiple comparisons was realized using the false discovery rate (FDR; *p.adjust* function) within each HII class (i.e. p-value adjusted within each HII class for four network metrics and the rarefied Shannon) or within each full core lake (i.e. p-value adjusted within each lake for the metrics calculated).

## RESULTS

### Landscape comparison of pre-Industrial to modern networks (top-bottoms)

**Fig. 3:**
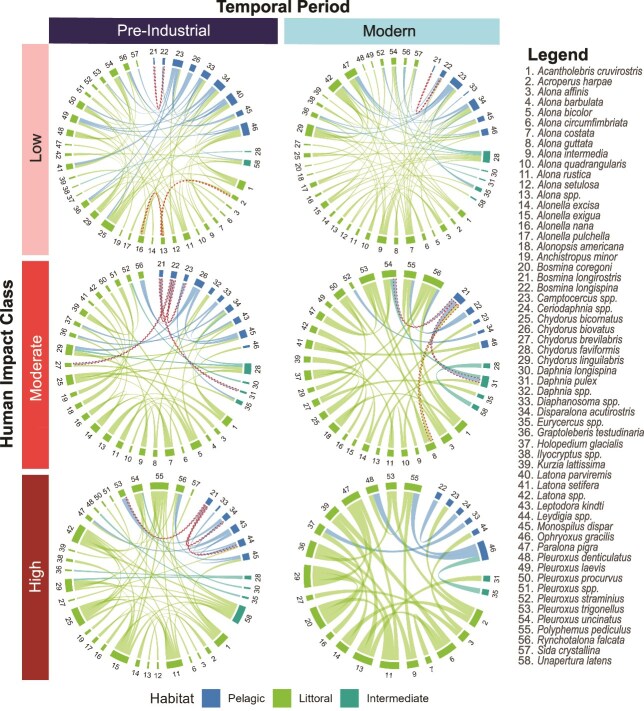
Chord diagrams of cladoceran network structures grouped by human impact class and time for lakes from the pre-Industrial and modern snapshot analyses (i.e. top-bottom comparisons). The numbers correspond to a specified cladoceran taxon. The links connecting taxa are only shown for species pairs where the Spearman correlation coefficient had a p-value < 0.01. The red dashed links signify negative significant Spearman correlation coefficients calculated between the taxa pair. The coloured links represent the habitat of the cladoceran taxa.

Our top-bottom analysis showed that the changes in network metrics varied with human impact. Lakes with low and moderate HII remained relatively well connected over time, while the network for the high impact sites lost links (Fig. 3). There were substantial decreases in network connectance, as well as in the number of nodes, edges, mean degree centrality, and skewness of degree distribution in high HII lakes over time (Table I). In contrast, low HII lakes showed only minor changes in nodes, edges, and mean degree centrality, with a slight decrease in modularity, and increase in skewness (Table I). Moderately impacted lakes also experienced modest losses in nodes and edges over time, but connectance remained stable (as observed with low HII lakes; Table I). In addition, their mean degree centrality decreased over time, while modularity and skewness increased (Table I).

**Table I:** *Metrics calculated from the networks shown in*  *Fig. 3**, based on subfossil cladocerans from the top-bottom comparisons, grouped by human impact class and temporal period*

Human Impact Class	Temporal Period	Nodes	Edges	Connectance	Modularity	Mean Degree Centrality	Skewness of Degree Distribution
Low	Pre-Industrial	43	73	0.081	0.47	3.40	0.85
	Modern	44	74	0.078	0.41	3.36	1.11
Moderate	Pre-Industrial	41	50	0.061	0.65	2.44	0.56
	Modern	35	37	0.062	0.72	2.11	0.70
High	Pre-Industrial	38	60	0.085	0.55	3.16	0.94
	Modern	27	27	0.077	0.78	2.00	0

**Fig. 4:**
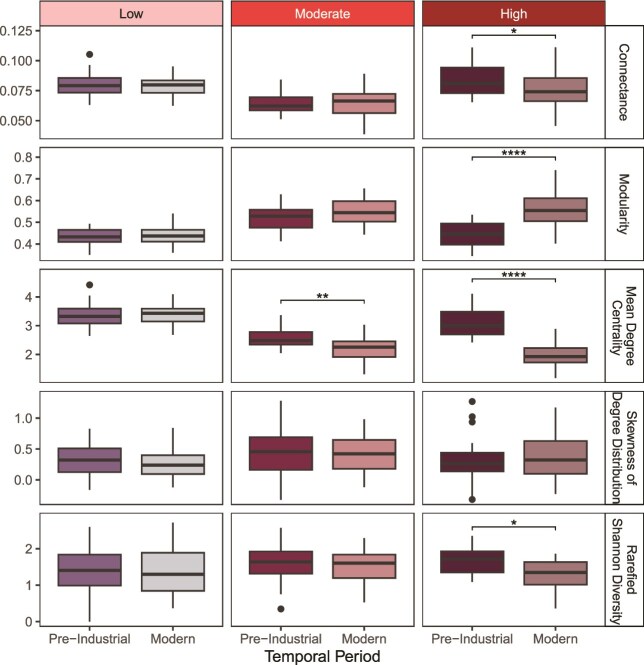
Boxplot of the distribution of network parameter values generated from random Erdős–Rényi network permutations (n = 30) to verify the patterns seen in observed networks from Fig. 3 and the rarefied Shannon diversity calculated for the top-bottom comparisons. One-way ANOVA and post-hoc Tukey’s HSD tests were used to test the significance of time within human impact classes (low, moderate, and high) for each network metric. A Mann-Whitney-Wilcoxon test was used to test the significance of the temporal change in rarefied Shannon diversity for low human impact class (n = 43), moderate (n = 39), and high (n = 19) at two time periods. Significance is denoted by an asterisk (^*^P < 0.05, ^**^P < 0.01, ^***^P < 0.001, ^****^P < 0.0001). The black points represent the outliers.

The random network simulations echoed most of the patterns detected in the original data (Fig. 4). For high HII lakes, we found that connectance (Tukey’s test: *P* = 0.027, FDR: *P* = 0.033), modularity (Tukey’s test: *P* < 0.001, FDR: *P* < 0.001), and mean degree centrality (Tukey’s test: *P* < 0.001, FDR: *P* < 0.001) were all significantly different between top-bottom comparisons for the random permutations. In contrast, random network metrics did not differ significantly among pre-Industrial and modern periods in low and moderate HII lakes, except the mean degree centrality metric in moderate HII lakes was different (Tukey’s test: *P* = 0.001, FDR: *P* = 0.006). Skewness was not significantly different over time for any of the HII categories. Rarefied Shannon diversity was only significantly different over the pre-Industrial and modern time period for high HII lakes (Mann-Whitney-Wilcon test: *P* = 0.008, FDR: *P* = 0.014).

### Full core analyses

**Fig. 5:**
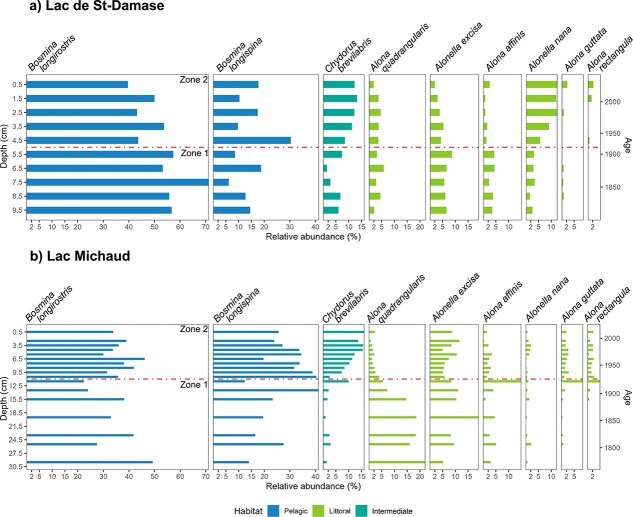
Stratigraphy of full core study lakes: (**a)** Lac de St-Damase, (**b)** Lac Michaud. Only cladocerans taxa with a relative abundance of at least 2%, as well as a cumulative relative abundance of at least 10%, are presented in the stratigraphy. The colours correspond to the type of habitat the cladoceran taxa. The red dashed lines delineate the zones based on the cluster analysis, where zone 1 is pre ~ 1920 CE and zone 2 is post ~ 1920 CE in both lake records.

**Fig. 6:**
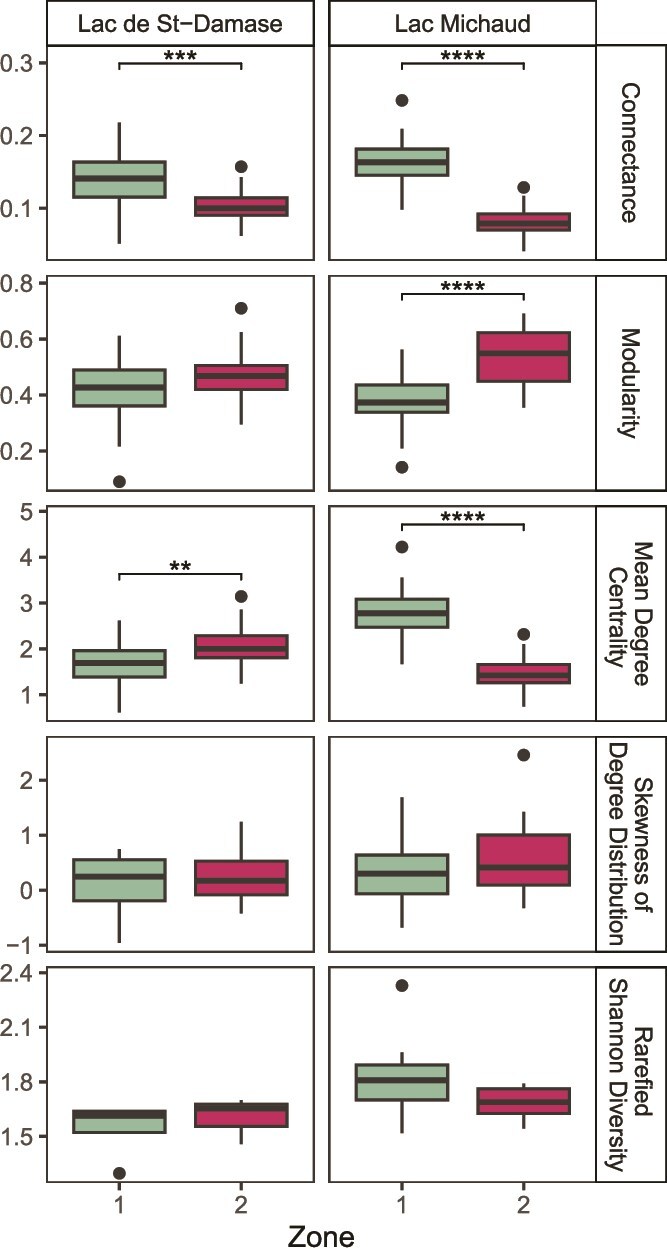
Boxplot of the distribution of network metrics and rarefied Shannon diversity values across zones of the two full cores. Network metric values were generated from random Erdős–Rényi network permutations (n = 30) to verify the patterns seen in observed networks from Fig. S3. One-way ANOVA and post-hoc Tukey’s HSD tests were used to test the significance of zone within each lake for each network metric. Rarefied Shannon diversity was calculated at each interval (or binned intervals when sedimentation rates were quite different) and then grouped into their respective zones. The Mann–Whitney-Wilcoxon test was used to test the significance of the rarefied Shannon diversity over the zones. Significance is denoted by an asterisk (^*^P < 0.05, ^**^P < 0.01, ^***^P < 0.001, ^****^P < 0.0001). The black points represent the outliers (that were still retained in our analyses).

There were marked changes in the relative abundance of taxa and in the network metrics over time in Lac de St-Damase. The cluster analysis identified significant zones based on the cladoceran assemblages from depths 5.5 to 9.5 cm (zone 1; ~ 1750–1916 CE) and 0.5 to 4.5 cm (zone 2; 1916–2017 CE; Fig. 5a). The relative abundance of *B. longirostris* was marginally higher in the pre-1916 CE cluster and stabilized in the more recent cluster. On the other hand, the relative abundance of *B. longispina* fluctuated substantially within zone 2, with a large peak ~ 1930 CE. The littoral taxa *Alona quadrangularis* and *Alonella excisa* had stable relative abundances with peaks before 1920 CE. *Alonella nana* and *Chydorus brevilabris* have been on the rise since the 1920s. Connectance and skewness of degree distribution decreased from zone 1 (pre-1916 CE) to zone 2 (post-1916 CE), whereas modularity and mean degree centrality increased over time (Table II, Fig. S3). Based on the random network simulations, connectance decreased significantly from zone 1 to zone 2 (Tukey’s test: *P* < 0.001, FDR: *P* < 0.001; Fig. 6). Mean degree centrality significantly increased (Tukey’s test: *P* < 0.001, FDR: *P* = 0.002; Fig. 6). Modularity (Tukey’s test: *P* = 0.04, FDR: *P* = 0.06; Fig. 6) had a tendency to increase. Skewness was not significantly different over the zones (Tukey’s test: *P* = 0.41, FDR: *P* = 0.41; Fig. 6). The rarefied (and binned) Shannon diversity was not significantly different across zones (Mann-Whitney-Wilcoxon test: *P* = 0.40, FDR: *P* = 0.41; Fig. 6).

**Table II:** *Metrics calculated from the networks shown in*  *Fig. 3(s)**, based on subfossil cladocerans from the full core analyses, grouped by significant temporal zones determined by CONISS of the cladocerans assemblage*

Lake	Zone	Zone Median Year	Nodes	Edges	Connectance	Modularity	Mean Degree Centrality	Skewness of Degree Distribution
Lac de St- Damase	1	1833	13	11	0.14	0.61	1.69	0.62
	2	1988	21	22	0.10	0.75	2.10	0.18
Lac Michaud	1	1857	18	25	0.16	0.40	2.78	1.00
	2	1970	19	15	0.09	0.76	1.58	0.74

Although we observed many of the same dominant taxa between the two full cores and a similar change point (circa 1920 CE) based on the cluster analysis, some key differences in assemblage dynamics were apparent in Lac Michaud (Fig. 5b). Based on the lake record, its sedimentation rate was much faster, allowing us to examine more sediment intervals. The cluster analysis identified significant zones from depths 11.5–29.5 cm (zone 1; ~ 1772–1924 CE) and 0.5–10.5 cm (zone 2; 1924–2017 CE; Fig. 5b). We detected clear directional changes in several littoral taxa, most notably *Alona affinis*, *Alona guttata*, and *C. brevilabris*; all being relatively rare since the 1780s followed by a small peak in 1920 CE. Since the 1920s, only *C. brevilabris* has increased substantially. In contrast, we found that *A. quadrangularis* decreased in relative abundance. Based on the network metrics calculated for each zone, we found that changes over time were consistent between lakes, with connectance and skewness of degree distribution decreasing and modularity increasing from zone 1 to zone 2 (Table II, Fig. S3). In contrast, mean degree centrality decreased over time. Based on the random network simulations, connectance decreased significantly from zone 1 to zone 2 (Tukey’s test: *P* < 0.001, FDR: *P* < 0.001; Fig. 6). Mean degree centrality (Tukey’s test: *P* < 0.001, FDR: *P* < 0.001; Fig. 6) and modularity (Tukey’s test: *P* < 0.001, FDR: *P* < 0.001; Fig. 6) increased significantly over the zones. Skewness of degree distribution was not significantly different over the zones (Tukey’s test: *P* = 0.11, FDR: *P* = 0.14; Fig. 6). We did not detect a significant difference in the rarefied Shannon diversity between zones (Mann–Whitney–Wilcoxon test: *P* = 0.19, FDR: *P* = 0.19; Fig. 6).

## DISCUSSION

Herein, we show substantial changes in the cladoceran network metrics over two different kinds of paleolimnological datasets that capture land-use gradients. In the top-bottom analysis of 101 lakes, we found that lakes with high human impact in their watersheds showed significant changes in their network parameters, particularly in the form of connectance and modularity shifts, whereas the same metrics stayed consistent in the low human impact lakes over time. In the full core analyses, we saw the same directional network changes over time for both study lakes. These observations align with our hypotheses that assemblages exposed to more disturbed conditions would demonstrate lowered connectance and increased modularity. In contrast, the mean degree centrality and skewness of degree distribution did not show consistent responses across the datasets, which may be attributed to the loss of generalist species.

### Consistent patterns in connectance and modularity

Our connectance results from assemblages experiencing disturbed conditions are consistent with other studies, where nutrient enrichment and temperature warming were found to negatively affect connectance of plankton and fish food webs in freshwater lakes (Kara *et al.,* 2013; Merz *et al.,* 2023; Bonnaffé *et al.,* 2024). We infer based on the design of our study lakes and findings from the literature that a decrease in connectance in the high impact lakes is caused by disturbances related to land-use, despite the uncertainty that connectance can consistently track environmental changes (Heleno *et al.,* 2012). Nonetheless, connectance has been at the forefront of the diversity-stability debate, questioning whether highly connected networks form very stable communities (McCann, 2000; Landi *et al.,* 2018). The uncertainty in connectance as a metric is likely caused by methodological differences among studies and must be standardized to fully understand its role in maintaining robustness and stability in the system but also be used alongside other network metrics (Landi *et al.,* 2018; Keyes *et al.,* 2024). Recent reviews have found that networks with high connectance tend to have greater robustness, as secondary extinctions are more constrained in highly connected networks (Dunne *et al.,* 2002; Keyes *et al.,* 2024). Hence, a loss of connectance may affect the stability and robustness of a community, rendering the community more vulnerable to the effects of disturbance. The consistency of our results across the top-bottom dataset along with the full cores provides a coherent signal and aligns with the land-use impact of our study sites.

We also detected a consistent increase in network modularity in high impact environments, both in the top-bottom comparison and in the full core analysis, relative to their pre-Industrial baselines. Our results contrast with earlier findings of Felipe-Lucia *et al.* (2020) who constructed synergistic networks of grasslands and forest ecosystems (including species, ecosystem functioning, and ecosystem services as nodes), and found mixed patterns in network connectance and decreases in modularity over an increasing land-use gradient. They attributed this decrease in modularity to the loss of highly specialized groups due to land-use changes. We postulate that modularity has increased in our more highly impacted lake networks owing to the loss of generalist taxa and their linkages that create subgroups in the network. For example, in the top-bottom comparisons, there were three connected subgroups in the pre-disturbed high HII lakes that were further separated in the modern assemblages. This includes a large subgroup with 12 taxa in the pre-disturbed high HII lakes. In modern times, this subgroup was reduced to five taxa. The taxa lost over time each had at least five links; lost taxa included *Alona rustica, Chydorus bicornatus, Chydorus linguilabris, Polyphemus pediculus, Unapterura latens, Latona spp., Alonella exigua,* and *Acantholebris cruvirostris,* while *A. excisa* and *Acroperus harpae* remained*.* There has been some evidence of specialists having success over generalists in the face of land-use and climate change (Sweeney and Jarzyna, 2022).

While high modularity may buffer extirpation and increase stability (May, 1972; Thébault and Fontaine, 2010; Stouffer and Bascompte, 2011; Landi *et al.,* 2018), certain conditions are required. Grilli *et al.* (2016) found that increased modularity can stabilize networks, but only when networks were composed of two equally-sized subunits, and when the overall mean interaction strength was negative; this makes it difficult to generalize the effect of modularity on network stability. In our data, we see subgroups in modern high HII lakes of the top-bottom comparisons that visually appear to be more equal in size than during pre-Industrial time. The potential stability provided by increased modularity may counter the potential loss of stability from lowered connectance found in high human impact sites. Although the modularity and connectance patterns may counter each other, it is also possible that each network metric targets different dimensions of stability (Donohue *et al.,* 2016): high modularity should buffer the effects of disturbances (Scheffer *et al.,* 2012), while high connectance networks may be more robust to secondary extinctions (Dunne *et al.,* 2002; Keyes *et al.,* 2024).

### Mixed patterns for mean degree centrality and skewness of degree distribution

Mean degree centrality decreased under high human impact in both the top-bottom comparison and the full core analysis of Lac Michaud, which has a relatively high HII (0.41). These results indicate that increases in watershed human activities decreased the average number of links per species present in the network. As land-use increases, the loss of links between taxa could result from dampened or interrupted associations with other species. On the other hand, Lac de St-Damase experienced increased mean degree centrality over time, despite a concomitant decrease in the number of links. Most taxa in the pre-1916 CE (i.e. zone 1) network had one or two links. However, in the post-1916 CE network (i.e. zone 2), *C. brevilabris* and *Chydorus faviformis* formed highly connected subgroups, resulting in a greater mean degree centrality. Hence over time, the species in Lac de St-Damase tended to increase associations with each other and perhaps become more generalist. There is also some evidence that taxa with greater degree centrality may have a greater probability of survival and hence greater persistence in the community (James *et al.,* 2012).

Skewness of degree distribution remained unchanged over time for all human impact levels in the random permutations, but there was a tendency for this metric to decrease in modern times in high HII lakes in the top-bottom comparison. Full cores showed a tendency to decrease in skewness over time, but the changes were not significant. The decrease in skewness of degree distribution with human land-use intensity is consistent with the results reported in earlier studies by Wang *et al.* (2019) and Li *et al.* (2022). Using diatom subfossil assemblages of several Chinese lakes at varying levels of land-use gradients, Wang *et al.* (2019) found that skewness of degree distribution became more negatively skewed as land-use development increased over time. Li *et al.* (2022) demonstrated that negative skewness of networks based on zooplankton collected from net tows in Chinese lakes were correlated with presence of omnivorous fish. Negatively skewed networks have greater generalist species and stronger connections between species (Wang *et al.,* 2019; Li *et al.,* 2022). Hence, this result suggests that the full core lakes have shifted towards generalist cladoceran species and are showing signs of being impacted by the effects of land-use. This is especially reflected in Lac de St-Damase as the degree mean centrality also increased over time due to having species with more links, which are assumed to be more generalist.

### Land-use signal in Lac de St-Damase and Lac Michaud

In our study the most predominant form of land-use was agricultural land, which is well known to cause enrichment in downstream waterbodies (Taranu and Gregory-Eaves, 2008; Soranno *et al.,* 2015). The time constrained cluster analysis delineated that the cladoceran community of Lac de St-Damase and Lac Michaud differed significantly pre and post ca. 1920 CE. The community change of Lac de St-Damase was coincident with a marked increase in sedimentary chlorophyll-*a* (including their degradation products, and measured through visible reflectance spectroscopy [VRS]), which Aucoin *et al.* (2025) attributed to enhanced agricultural activities in the watershed. Indeed, in 2017, the watershed of Lac de St-Damase consisted of 24% agriculture and pastures. In Lac Michaud, the sedimentary chlorophyll-*a* (and degradation products) time series (measured through VRS) was more complex, yet showed the greatest increases post-1950 (Aucoin *et al.,* 2025), with as much as 48% of the watershed devoted to agriculture or pastureland by 2017. In addition, geochemical erosion indicators suggest an urbanization and deforestation signal in Lac Michaud watershed around 1913 CE (Aucoin *et al.,* 2025). Taken together, land-use processes (such as agriculture, urbanization, and deforestation) likely influenced nutrient inputs, explaining the significant change in cladoceran assemblage ~1920s.

### Network ecology complements diversity metrics

Using the same top-bottom dataset, Paquette *et al.* (2022) found that alpha diversity (specifically Shannon diversity) decreased significantly in the high HII lakes over time, which we also observe in this study with the rarefied values (see Figs. S4, S5, S6 for the community composition of lakes within each HII class). By adopting a network lens, we found that connectance decreased, while modularity increased over time, especially in environments with intensive human activity. Temporal beta-diversity across lakes of different human impact classes showed a tendency to increase, with a pronounced change in five of the 19 high HII lakes (Paquette *et al.,* 2022). Considering these results together with ours, we infer that species replacement may be occurring, but this replacement does not appear to be stabilizing because of a decrease in connectance over time. With the addition of network parameters, we gained more insight into the underlying mechanisms of species loss.

For both full core lakes, Lac de St-Damase and Lac Michaud, we detected similar changes in network metrics over the zones: connectance significantly decreased, while modularity increased (only significant in Lac Michaud). Mean degree centrality likewise changed significantly over the zones, but in opposite directions among both full core lakes. Rarefied Shannon diversity (and also binned into equal time units for Lac de St-Damase) did not significantly shift over the stratigraphic zones for both full core lakes across zones. As there were significant changes in network metrics over the zones, but no significant changes in the rarefied Shannon diversity over the same periods, we have evidence that network metrics may be more sensitive to detect shifts in cladoceran community than Shannon diversity. Even if alpha diversity remains stable, the dynamics within the community may not be stable as stressors or disturbances such as land-use intensify. This mirrors what was postulated by Taranu *et al.* (2023), that network parameters can be more sensitive in detecting community changes and thereby, complement diversity metrics.

### Limitations

Common to all ecological network approaches (including those based on direct observations of biotic interactions), associations may arise due to environmental filtering, where species that co-occur have similar environmental niches. As such, the co-occurrences we observed herein may, at least in part, be a result from shared environmental preferences and settlement history (Freilich *et al.,* 2018). Thus, shifts in co-occurrence network structure with human impacts to lake watersheds can reflect both changes in potential interactions and/or environmental conditions. Therefore, declining connectance over time in lakes with more watershed land-use can be interpreted as species no longer sharing similar abundance dynamics and perhaps becoming disconnected as environmental conditions change. A promising area for future studies would be to disentangle the environmental influence component of species associations within correlation-based co-occurrence networks. Specifically, by incorporating analyses of species’ co-responses and niche dynamics, one can further tease apart the correlations found between taxa pairs due to abiotic filtering vs potential biotic interactions (Taranu *et al.,* 2021). Other factors may further explain species associations, such as spatial autocorrelation related to a shared geography, as well as indirect associations, where species may associate due to a common interaction (or association) with another species (Carr *et al.,* 2019).

Future studies could also consider multiple trophic level networks to more accurately represent entire food webs. With paleolimnology, analyses of DNA preserved in lake sediments can be used to detect the abundances of a wide range of taxa over decades to centuries and provides potentially rich time series that better characterize the full ecological networks of lakes (Gregory-Eaves *et al.,* 2023). Finally, as only land-use changes were considered explicitly in this study, expanding the pool of sites should make it possible to disentangle network responses to land-use from those driven by climatic change, species richness, or regional context.

## CONCLUSION

Here, we show how zooplankton networks have changed over time in lakes due to the cumulative effects associated with watershed land-use using two paleolimnological approaches. With the top-bottom comparisons, we showed these changes on a national scale using cladoceran assemblages from 101 pan-Canadian lakes. We also observed similar patterns with the full core approach, whereby cladoceran assemblage dynamics were evaluated at a high temporal resolution in two lakes. The constructed networks represent an important starting point that can be further probed to define the relative strengths of environmental preferences of taxa and biotic interactions underlying the observed patterns. In addition, future studies can extend this work by expanding the networks to include other trophic levels, so they are more representative of realistic food webs.

## Supplementary Material

Supplementary_material_Sept2025

## Data Availability

Data for the relative abundances of cladoceran subfossils using the top-bottom approach over 101 pan-Canadian lakes, as well as their respective human impact index, are found in Paquette *et al.* (2022), with data available at the Zenodo data portal (https://doi.org/10.5281/zenodo.4701262). The relative abundances of cladoceran subfossils of Lac de St-Damase and Lac Michaud are described in Aucoin *et al.* (2025) and are publicly available in this Zenodo data portal (https://doi.org/10.5281/zenodo.15986788).
