# Supplementary material for: Temporal dynamics of ecological networks: deciphering changes in cladoceran assemblages over the past ~ 150 years in response to land-use development
Source: J Plankton Res. 2025 Sep 20;47(5):fbaf047. doi: 10.1093/plankt/fbaf047 (PMC12449760; doi:10.1093/plankt/fbaf047)
Supplement: Supplementary_material_Sept2025 [file supplementary_material_sept2025.docx]

Supplementary material**
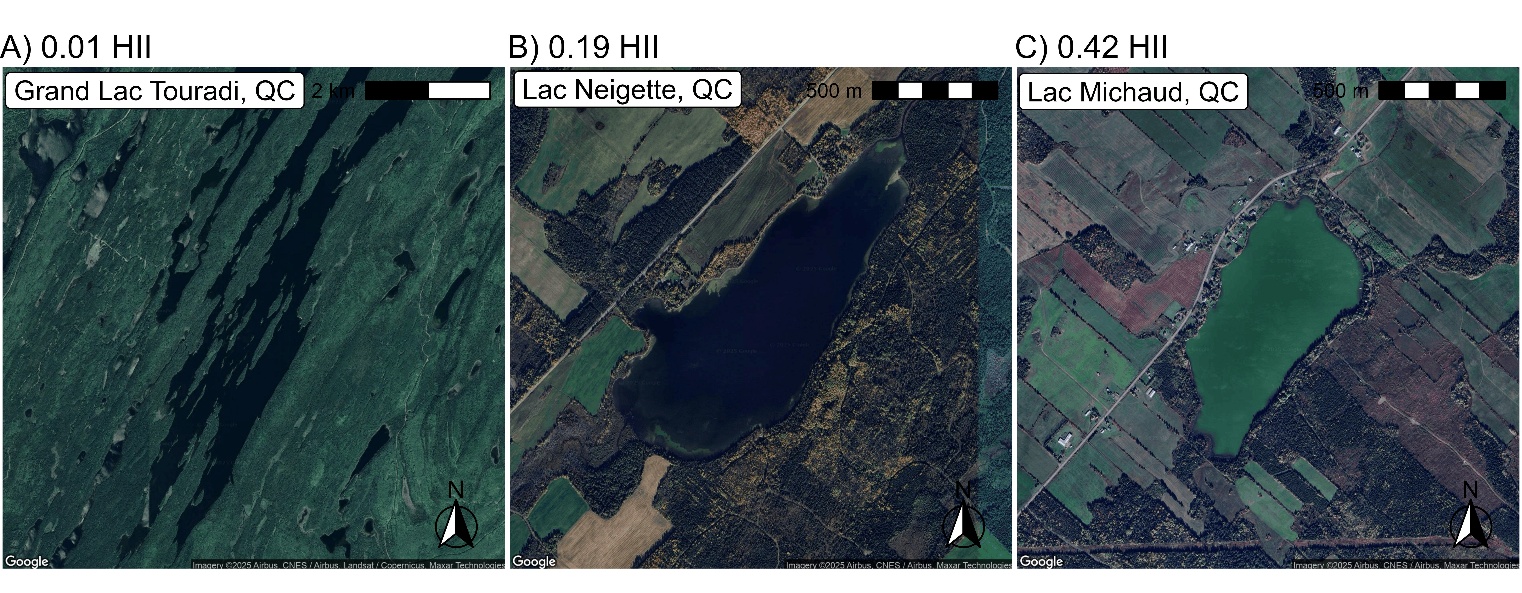
**

**Figure S1.** Satellite view of three Quebec lakes in the Atlantic highlands ecozone of Canada each with three differing levels of human impact in their respective watersheds, as determined by the LakePulse Network. A) Grand Lac Touradi (48.1, -68.7) has a low HII (0.01) and is surrounded by natural landscapes. B) Lac Neigette (48.3, -68.4) has a moderate HII (0.19) and is immediately surrounded by agricultural fields and forests. C) Lac Michaud (48.6, -67.8) has a higher human impact index (HII; 0.42) and the lake is immediately surrounded by agricultural fields.

**
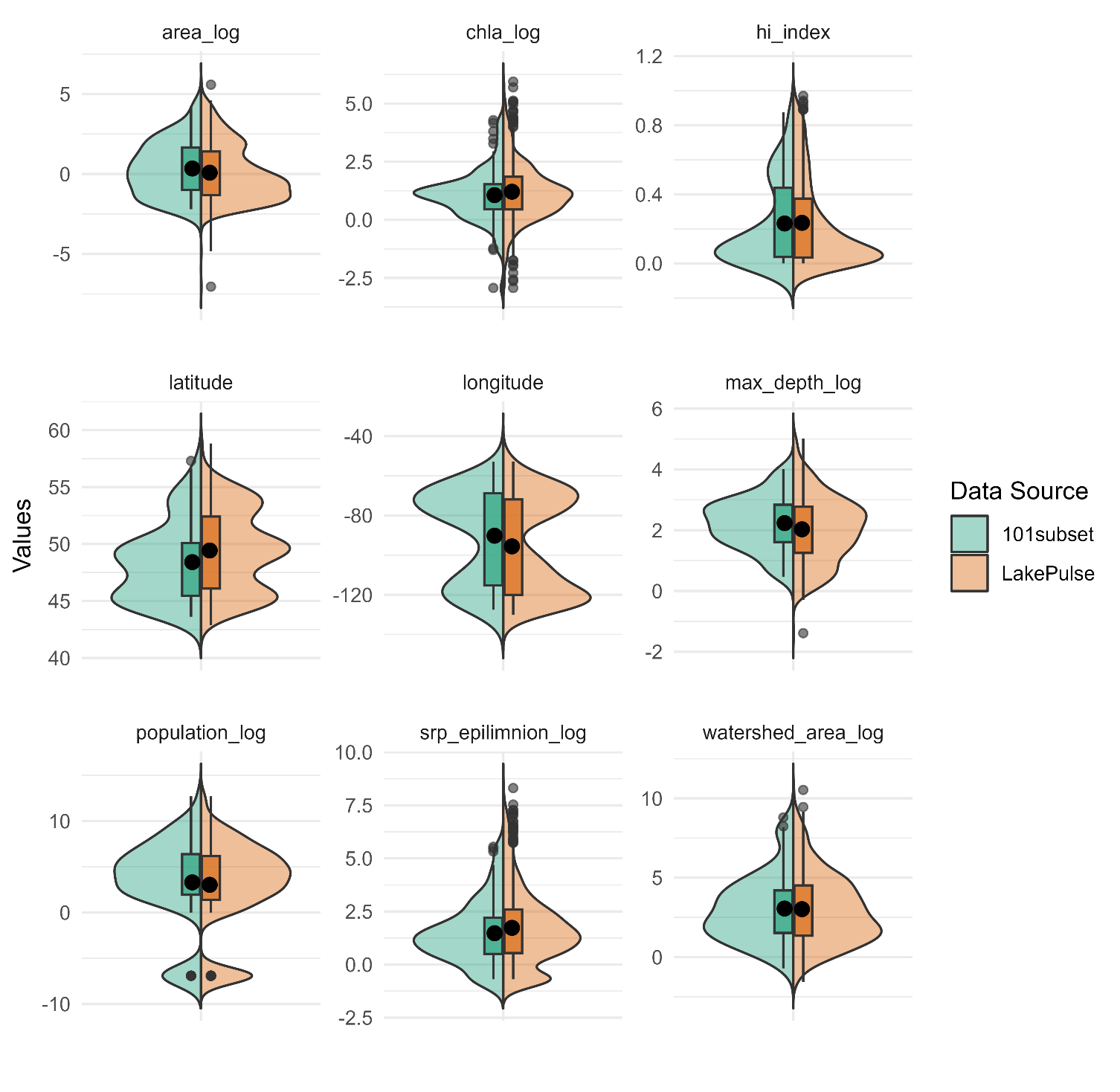
**

**Figure S2.** Split violin plots comparing the distribution of the 101 top-bottom lake subset to the 606 lake LakePulse dataset over various limnological variables.


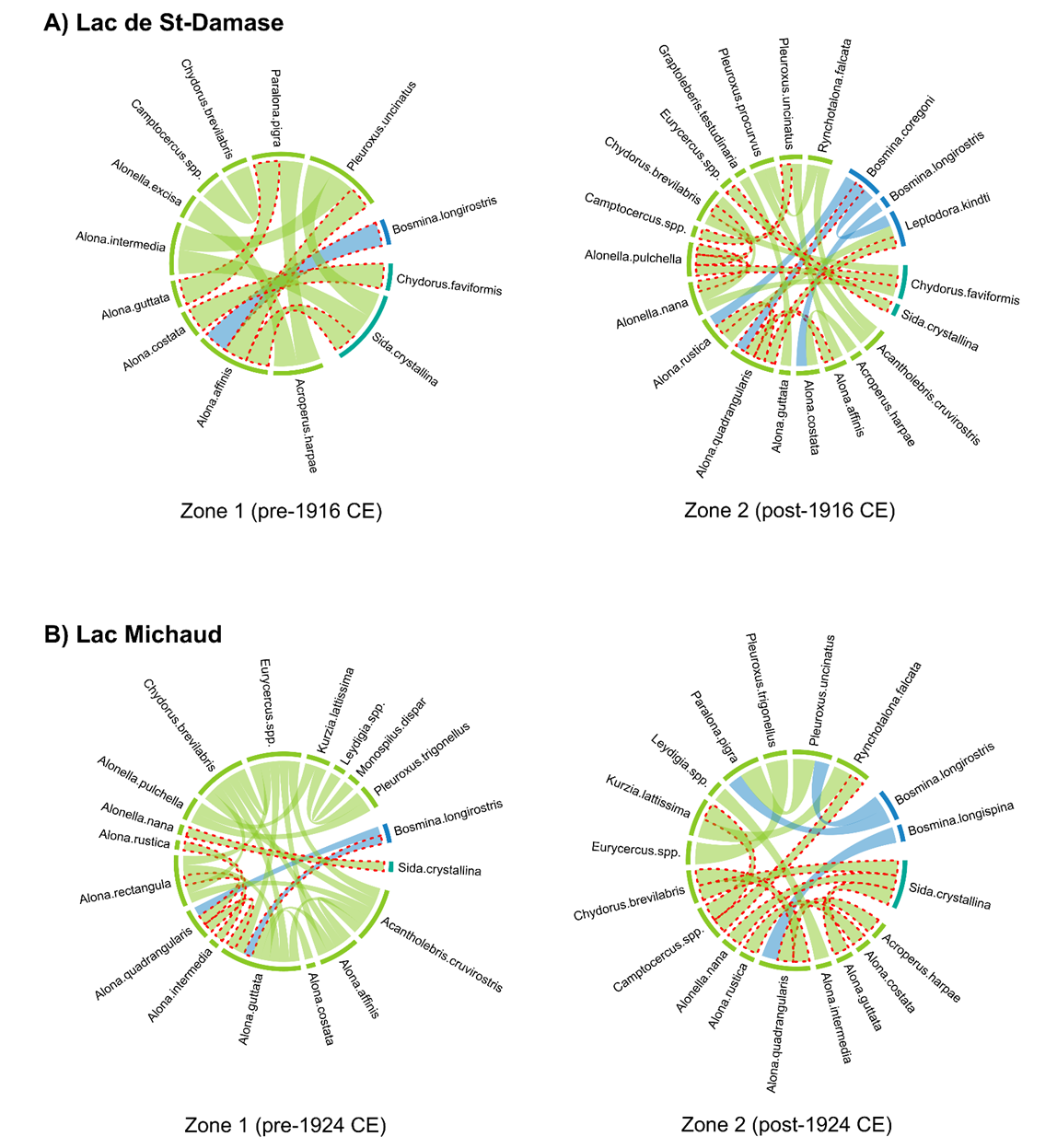


**Figure S3.** Chord diagrams of cladocerans network structures for zone 1 and 2 for each full core lakes A) Lac de St-Damase and B) Lac Michaud. The links connecting species pairs correspond to their Spearman correlation coefficient, which had a p-value less than or equal to 0.05. The red and dashed lines represent negative Spearman correlation coefficient. The colours of the links represent the habitat of the cladoceran taxa.

**
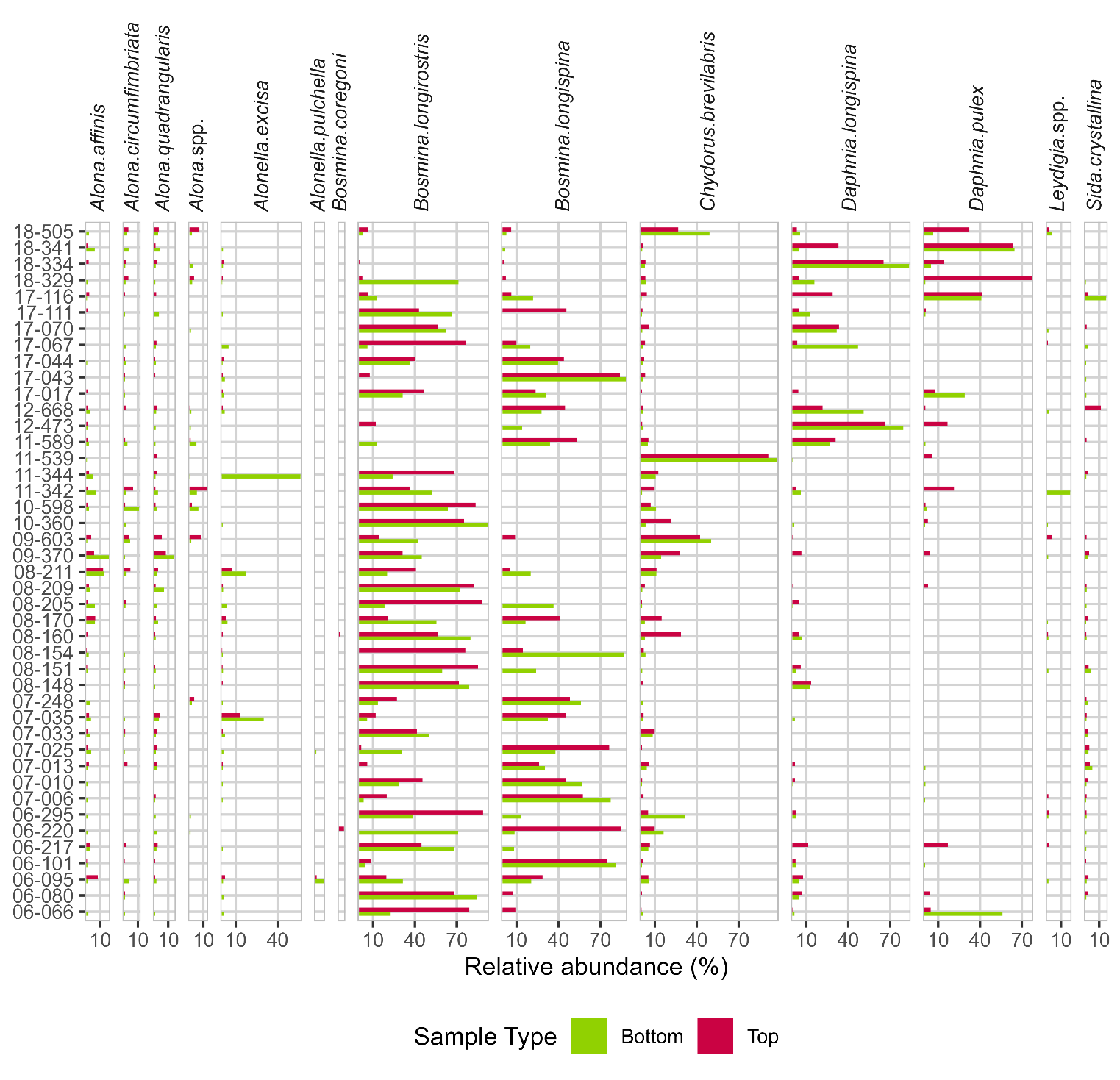
Figure S4.** Plots depicting the changes in the relative abundances of the cladoceran assemblage of the top and bottom intervals of the low HII lakes (n = 43). The y-axis is LakePulse lake codes, while the x-axis is the cladoceran taxa with its relative abundances. Only taxa with at least 15% relative abundance in either the top or bottom interval of lakes across all human impact classes are shown.

**
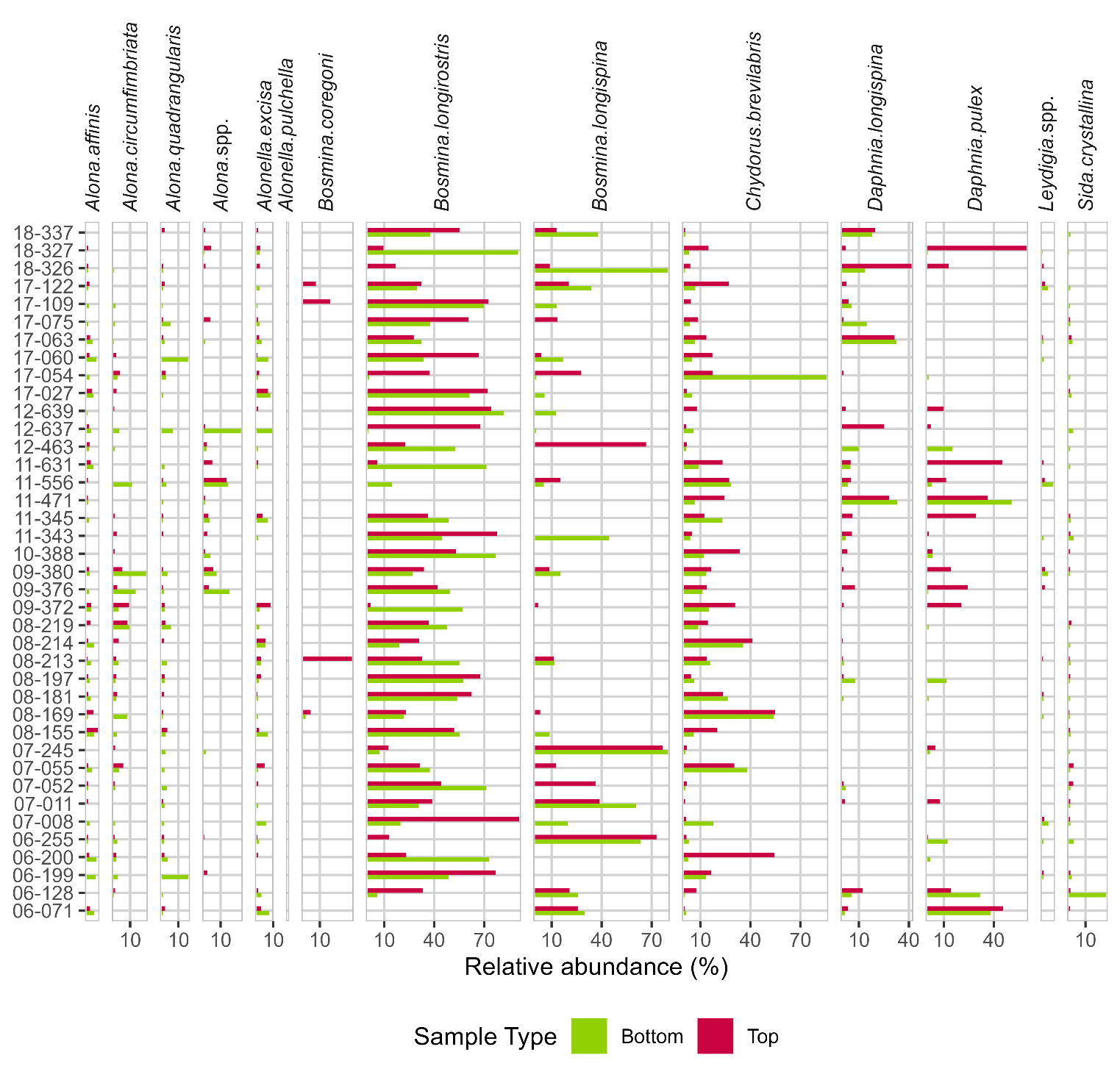
Figure S5.** Plots depicting the changes in the relative abundances of the cladoceran assemblage of the top and bottom intervals of the moderate HII lakes (n = 39). The y-axis is LakePulse lake codes, while the x-axis is the cladoceran taxa with its relative abundances. Only taxa with at least 15% relative abundance in either the top or bottom interval of lakes across all human impact classes are shown.

**
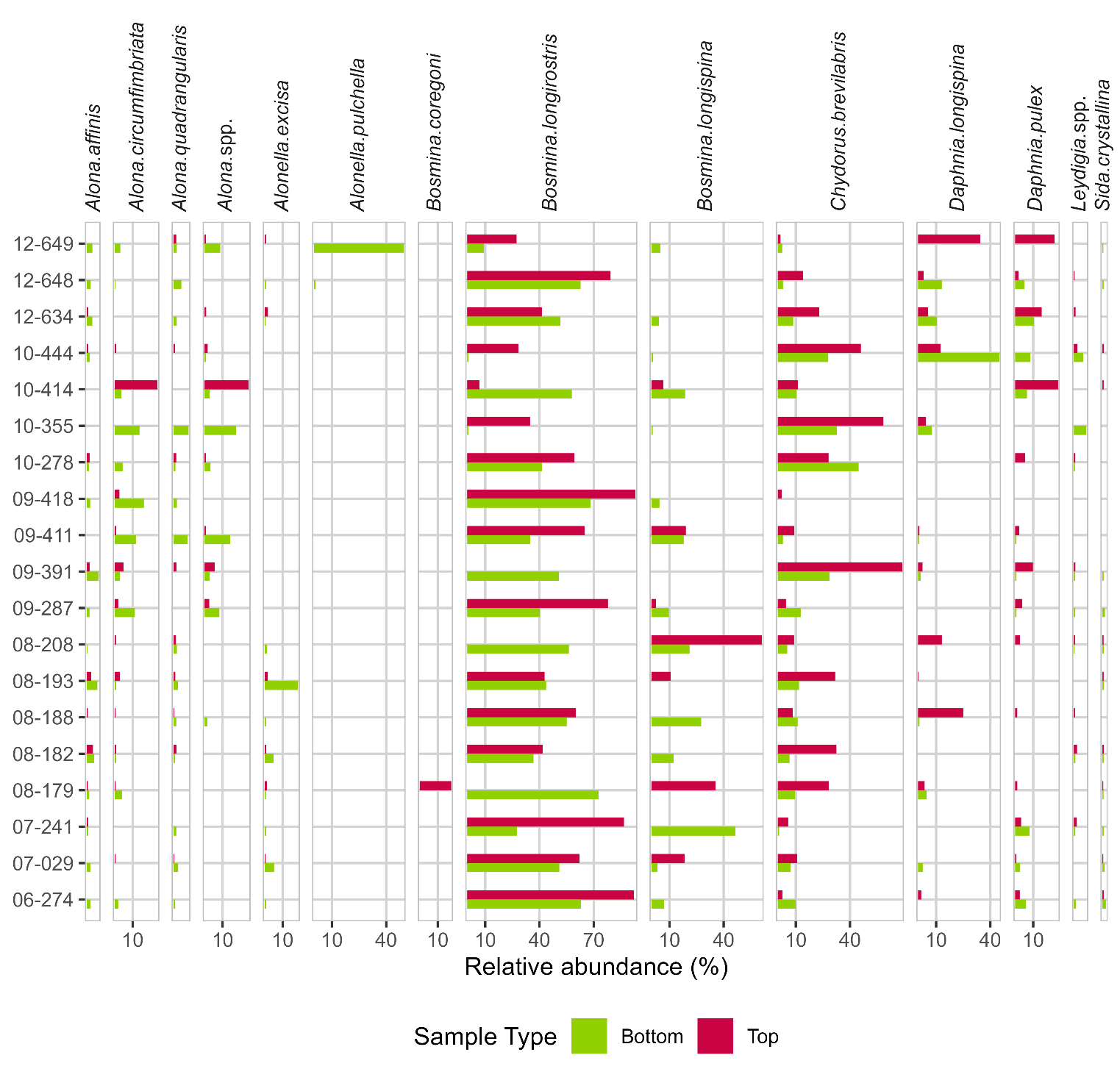
Figure S6.** Plots depicting the changes in the relative abundances of the cladoceran assemblage of the top and bottom intervals of the high HII lakes (n = 19). The y-axis is LakePulse lake codes, while the x-axis is the cladoceran taxa with its relative abundances. Only taxa with at least 15% relative abundance in either the top or bottom interval of lakes across all human impact classes are shown.
